# Effect of thermal annealing Super Yellow emissive layer on efficiency of OLEDs

**DOI:** 10.1038/srep40805

**Published:** 2017-01-20

**Authors:** Samantha Burns, Jennifer MacLeod, Thu Trang Do, Prashant Sonar, Soniya D. Yambem

**Affiliations:** 1 School of Chemistry Physics and Mechanical Engineering, Queensland University of Technology (QUT), Brisbane QLD 4000, Australia; 2 Institute for Future Environments, Queensland University of Technology (QUT), Brisbane QLD 4000, Australia; 3 Institute of Health and Biomedical Innovation, Queensland University of Technology (QUT), Kelvin Grove, Queensland 4053, Australia

## Abstract

Thermal annealing of the emissive layer of an organic light emitting diode (OLED) is a common practice for solution processable emissive layers and reported annealing temperatures varies across a wide range of temperatures. We have investigated the influence of thermal annealing of the emissive layer at different temperatures on the performance of OLEDs. Solution processed polymer Super Yellow emissive layers were annealed at different temperatures and their performances were compared against OLEDs with a non-annealed emissive layer. We found a significant difference in the efficiency of OLEDs with different annealing temperatures. The external quantum efficiency (EQE) reached a maximum of 4.09% with the emissive layer annealed at 50 °C. The EQE dropped by ~35% (to 2.72%) for OLEDs with the emissive layers annealed at 200 °C. The observed performances of OLEDs were found to be closely related to thermal properties of polymer Super Yellow. The results reported here provide an important guideline for processing emissive layers and are significant for OLED and other organic electronics research communities.

Organic light emitting diodes (OLEDs) are highly sought after for display technology and solid state lighting. High colour contrast^1^, flexibility^2^, wide viewing angles^3^ and printability^4^ make OLEDs highly desirable for display technologies such as screens for mobile phones and curved TVs. OLED is also an upcoming technology for solid state lighting^5^, since it can provide uniform illumination over a large area^6^ and offers the possibility for complex lighting designs and patterns. OLEDs have also been successfully implemented in wearable sensing devices for applications in medicine and robotics^7^,^8^.

An attractive attribute of OLED technology is the solution processibility of constituent layers, since solution processable OLEDs have the potential to be printed into complex designs and shapes of light emitting areas/pixels. The performance of solution processable OLEDs is influenced to a great extent by processing conditions of the emissive layer, such as solvents^9^, thermal treatment^10^ etc., since these conditions influence the morphology of the films, the interfaces between consecutive layers and the optical and electrical properties of the device^11^. Liu *et al*. showed that thermal treatment of the emissive layer homopolymer poly[2-methoxy-5-(2-ethylhexyloxy)-1,4-phenylenevinylene] (MEH-PPV) influences the efficiency of OLEDs extensively^12^. With an increasing annealing temperature of MEH-PPV, the current efficiency of the OLEDs increased, reaching a maximum at the optimum temperature beyond which the efficiency dropped. A similar trend in luminance was observed by Jokinen *et al*. for OLEDs with a poly(9,9-di-*n*-octylfluorenyl-2,7-diyl) (PFO) emissive layer^10^. Ahn *et al*. reported a different result showing no difference in external quantum efficiency (EQE) between OLEDs with non-annealed and annealed MEH-PPV films^13^. The influence of thermal annealing of doped emissive layers such as poly(9,9-dioctylfluorene-*alt*-benzothiadiazole) (F8BT) in PFO and blended layer emissive layers such as MEH-PPV and 2,7-bis[2-(4-*tert*-butylphenyl)-1,3,4-oxadiazol-5-yl)-9,9-dihexylfluorene (DFD) on performance of OLEDs have also been reported by Jokinen *et al*.^10^ and Ahn *et al*.^13^, respectively. The luminance for PFO:F8BT blend OLEDs and EQE for MEH-PPV:DFD blend OLEDs decreased upon annealing the blended emissive layer. These reports suggest that there is an optimum temperature for processing the emissive layer and identifying this point is of key importance to optimize device performance. The effect of processing conditions on efficiency and performance is also seen in other organic electronic devices such as organic photovoltaics and organic field effect transistors^14^,^15^.

Among solution-processable conjugated polymer emissive layers, copolymer Super Yellow is one of the most widely used emissive layers in organic light emitting devices including OLEDs^3^,^16^,^17^,^18^,^19^. It has been shown that different solution processing techniques for fabricating Super Yellow thin films have limited influence on its photophyical and charge transporting properties^20^. However, the effect of thermal annealing Super Yellow thin films during OLED fabrication has not been systematically documented. As such, research reports in organic light emitting devices using polymer Super Yellow as emissive layer show a wide range of thermal treatment, from non-annealed films to annealing at temperatures up to 200 °C^18^,^21^,^22^,^23^,^24^. A brief summary of different annealing temperatures is listed in Table S1 (Supplementary Information). Furthermore, there is limited knowledge of thermal properties of conjugated polymer Super Yellow.

In this work, we have done a systematic investigation of the effect of annealing polymer Super Yellow films on the efficiency of OLEDs. Non-annealed Super Yellow films and films annealed at 50 °C, 100 °C, 150 °C and 200 °C were investigated. The results show an optimum annealing temperature of 50 °C, which produced OLEDs that reached a maximum EQE of 4.09%. This is significantly higher than EQE of OLEDs with Super Yellow emissive layer annealed at 200 °C, which had a maximum efficiency of only 2.72%. The thermal properties of polymer Super Yellow and the optical and morphological thin film properties were investigated to explain the trend observed in OLED performance with respect to annealing temperature.

## Results

### OLED Performance

**Figure 1:**
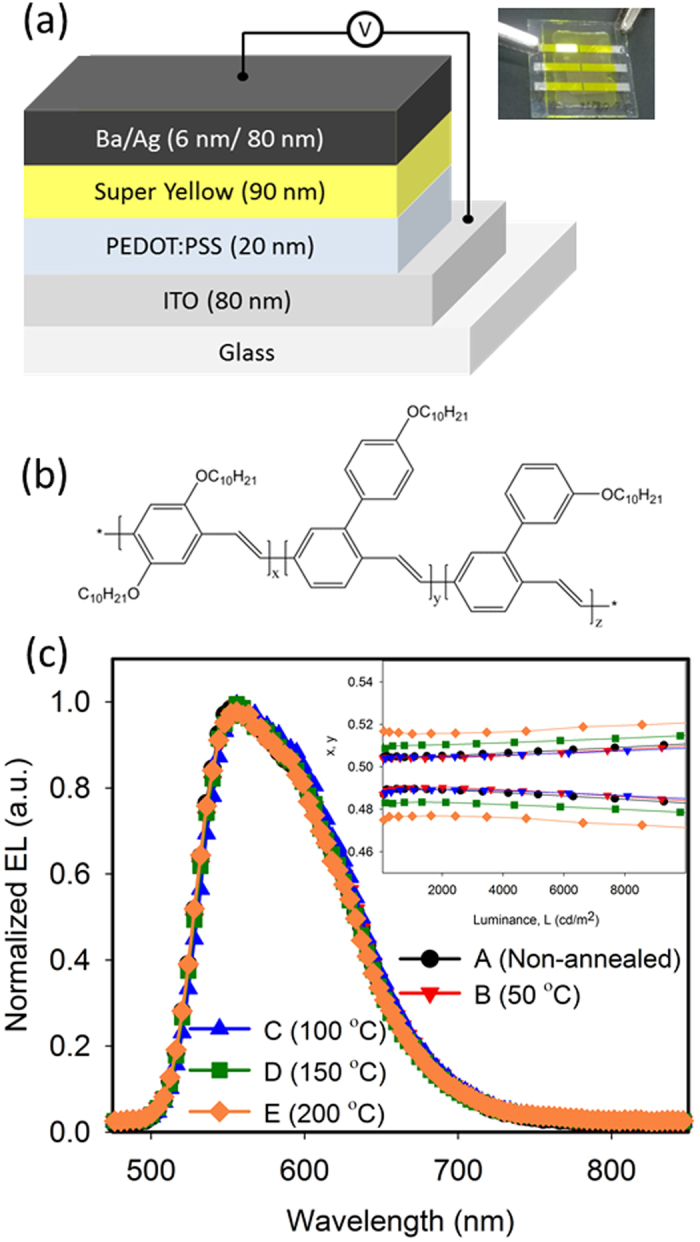
(**a**) Device structure of OLEDs in this study. Inset: picture of an OLED pixel. (**b**) Chemical structure of polymer Super Yellow. (**c**) Normalized electroluminescence (EL) for OLEDs of each category. Inset shows CIE x and y co-ordinates for OLEDs of all sets at different brightness.

To investigate the effect of annealing Super Yellow films on efficiency of OLEDs, we fabricated five sets of OLEDs, sets A, B, C, D and E, with different annealing temperatures. A schematic of the device structure is shown in Fig. 1(a). The thermal annealing step was carried out after deposition of the Super Yellow layer. Set A has non-annealed Super Yellow films while sets B, C, D and E have Super Yellow films annealed at 50 °C, 100 °C, 150 °C and 200 °C, respectively. The chemical structure of polymer Super Yellow is shown in Fig. 1(b). In our devices, poly(3,4-ethylenedioxythiophene):polystyrene sulfonate (PEDOT:PSS) was used as a hole transport layer and Ba was used as an electron injection layer.

Normalized electroluminescence (EL) spectra of all sets of OLEDs are shown in Fig. 1(c). There is no visible influence on the EL spectra by annealing Super Yellow films at different temperatures. CIE co-ordinates of all sets of OLEDs at different brightness are shown in inset of Fig. 1(c), which shows little or no variation in emitted colour among the OLEDs. Broadening of EL spectrum and/or shifts in the emission peak upon annealing the emissive layer have been reported for other polymer systems^10^,^12^,^13^.

**Figure 2:**
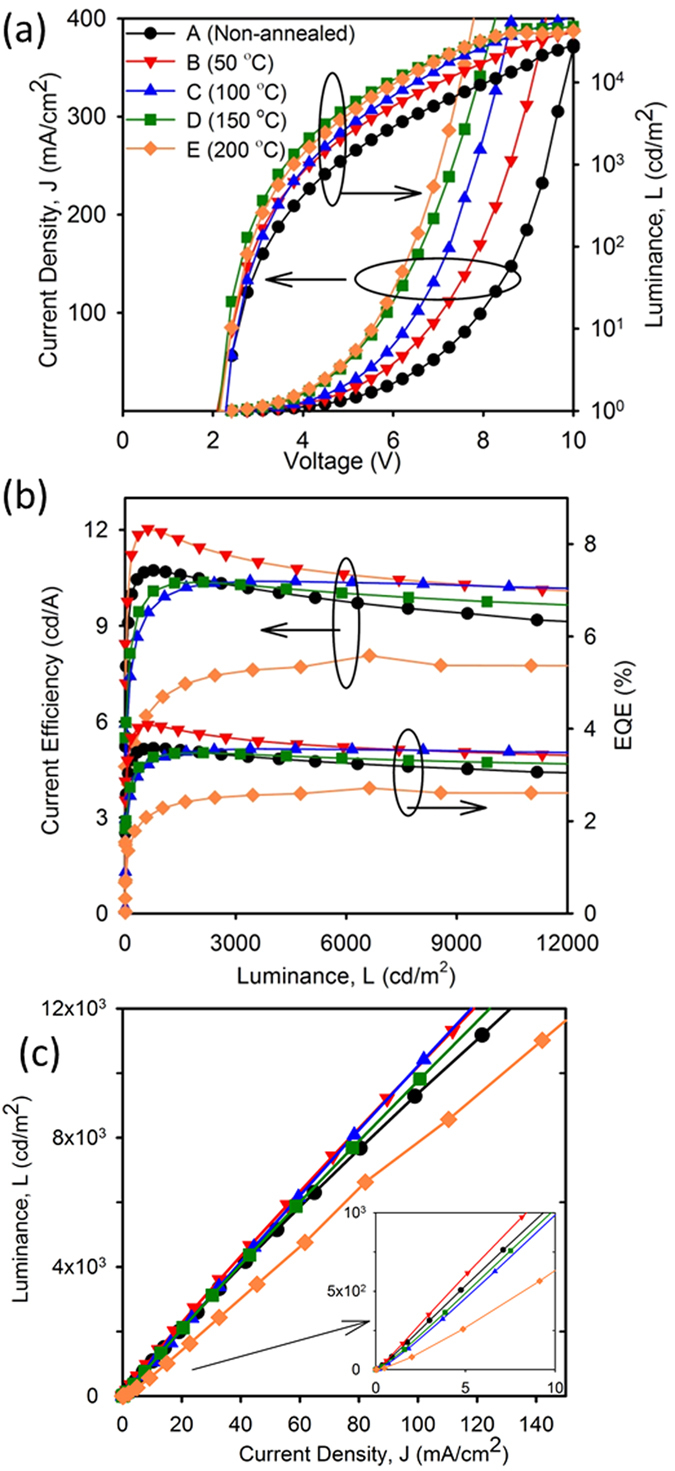
(**a**) Current density and luminance with respect to voltage for the best device in each category. (**b**) Current efficiency and external quantum efficiency for the best device in each category. (**c**) Luminance with respect to current density for OLEDs corresponding to Fig. (**a**). Inset shows magnification of the plot at low current density. Legend of (**a**) is valid for (**b**) and (**c**).

Current density and luminance with respect to voltage for the best device in each set are shown in Fig. 2(a). As seen in the figure, there are variations in both current density and luminance when the Super Yellow films are annealed at different temperatures. The current density increases monotonically at all voltages from set A to set D. This indicates increased bulk conductivity arising from a denser film of Super Yellow. Gather *et al*. have reported a similar observation of increasing current density with increase in annealing temperature for white OLEDs^25^. The trend of increasing current density with higher annealing temperature changes slightly with set E which has almost the same current density as set D at lower voltages but higher current density at higher voltages. A similar trend to current density is seen for luminance with respect to voltage. Luminance increases from sets A to D but for set E the luminance starts decreasing. The turn on voltages are given in Table S1 (Supplementary Information). The average turn on voltage of all sets varies between 2.10 V to 2.28 V. Current efficiency and EQE plots against luminance for the best devices are shown in Fig. 2(b). The trend in current efficiency and EQE is the same for all sets of OLEDs. The difference between efficiencies is more pronounced at lower brightness. Set B has the highest efficiency reaching a maximum current efficiency of 12.03 cd/A and a maximum EQE of 4.09% and set E has the lowest efficiency with maximum current efficiency and EQE of 8.07 cd/A and 2.72%, respectively. For sets A and B, the efficiency reaches a maximum at lower brightness and starts rolling off, while for sets C, D and E the efficiency rises at a slower rate and holds steady or has a lower roll off. Averages of maximum efficiencies are given in Table S1 and averages of current efficiencies and EQEs at 100 cd/m^2^, 1000 cd/m^2^ and 10000 cd/m^2^ are given in Table S1.

From Fig. 2(a) and (b), we see that current density is highest for set E, however set D has the highest brightness and the efficiency is maximum for set B. This implies that the OLED with the highest current density is not necessarily the brightest and the brightest OLED does not necessarily have the highest efficiency. The trend in efficiency is easier to see from Fig. 2(c), which is a plot of brightness against current density. Higher luminance at the same current density implies higher efficiency. The inset of Fig. 2(c) is a magnification of the plot at lower current density. From the difference in luminance at the same current density between the different sets of OLEDs, we can infer that there are intrinsic properties of polymer Super Yellow that are affected by thermal annealing, and that these play a role in the overall performance of the device.

### Super Yellow Properties

**Figure 3:**
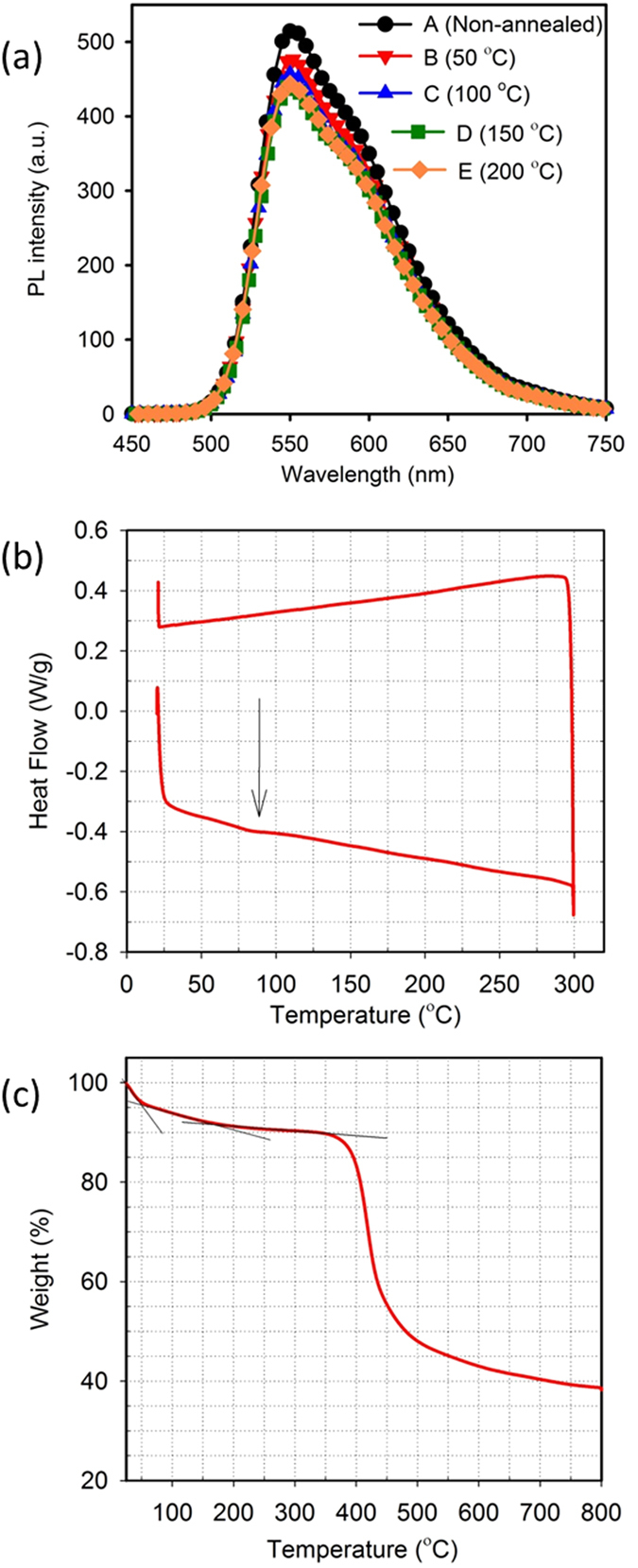
(**a**) Photoluminescence (PL) spectra of non-annealed and annealed Super Yellow films. (**b**) Differential scanning calorimetry curve of polymer Super Yellow. The arrow indicates T_g_ of the polymer. (**c**) Thermogravimetric analysis of polymer Super Yellow. The lines are inserted as a guide for the eye.

Photoluminescence (PL) intensities were measured for non-annealed and annealed Super Yellow thin films to investigate the effect of annealing temperature on photophysical properties. As shown in Fig. 3(a), the non-annealed film has higher intensity than all annealed films. The intensities gradually decrease with increasing annealing temperature up to 150 °C, beyond which there is no change in intensity. There is no variation in the shape of the spectra with annealing temperatures. A previous report on PPV based polymers^12^ demonstrated that the shape of PL spectra for PPV derivatives with large side groups is not affected by annealing temperature. Since Super Yellow is a PPV based co-polymer with big side groups, our results are in agreement with this report. Therefore, even though there is a difference in the intensity of emission, the emitted colour will remain stable. This is reflected in the EL for all sets of OLEDs, as well as in the constancy of the observed colour co-ordinates.

For a deeper understanding of thermal properties of polymer Super Yellow, we performed differential scanning calorimetry (DSC) and also carried out thermogravimetric analysis (TGA). Results of DSC and TGA are shown in Fig. 3(b) and (c), respectively. From the DSC scan we see that the glass transition temperature (T_g_) of polymer Super Yellow is around 83 °C. The T_g_ obtained from our DSC scan is significantly below that reported earlier for T_g_ of Super Yellow polymer (~150 °C)^26^. Given that molecular weight of a polymer plays a critical role in T_g_, it is highly like there will be variations in T_g_ between different batches of the polymer. For Super Yellow used in this study, we determined the molecular weight by gel permeation chromatography (GPC) which showed a number average molecular weight (*M*n) of 184.3 kDa with polydispersity index (PDI) of 1.38. Gel permeation chromatogram is shown in Fig. S1 (Supplementary Information).

On the other hand, TGA showed that polymer Super Yellow starts degrading at lower temperatures and has lost almost 4% of its weight by 50 °C. From Fig. 3(c), we see three distinct regions of weight loss before 350 °C. The first region, which extends approximately up to ~50 °C, has a steeper slope than the second region, which extends up to ~175 °C. The third region extends up to ~350 °C, and again has a shallower slope as compared to the second region. By 200 °C, which is the highest temperature in our study, the polymer has lost ~9% of its weight. This weight loss might be due to breaking of the long alkoxy chain substituted on the benzene ring and vinylene bond present between the phenylene units. Beyond 350 °C the polymer starts degrading rapidly on a path to complete degradation.

**Figure 4:**
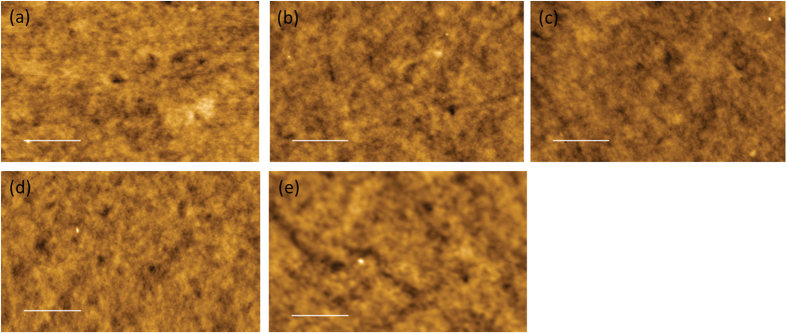
AFM images of glass/ITO/PEDOT:PSS/Super Yellow films: (**a**) non-annealed and annealed at (**b**) 50 °C (**c**) 100 °C (**d**) 150 °C and (**e**) 200 °C. The scale bar denotes 1000 nm. RMS roughnesses of the films are in the range of 0.5 nm to 1 nm.

To determine the influence of annealing temperature on the surface morphology of Super Yellow thin films in the device, atomic force microscopy (AFM) was performed on glass/ITO/PEDOT:PSS/Super Yellow films that were annealed at different temperatures. Typical AFM images of all films are shown in Fig. 4. We see little or no difference in the features on non-annealed and annealed films. Since the longer decyloxy side chains substituted on phenylene-vinylene conjugated backbone of polymer Super Yellow will prevent aggregation, no significant changes in morphology are expected upon thermal annealing the films. AFM scans at different locations on the films showed little or no difference in roughness of the films. The measured RMS roughnesseses of the films are all in the range from 0.5 to 1.0 nm (Fig. S2, Supplementary Information). The smooth, irregular AFM morphology clearly reveals that the polymer is amorphous by nature and there is no presence of any ordering.

## Discussion

From the results obtained, we can infer that both the electrical and optical properties of polymer Super Yellow are affected by thermal treatment, which consequently affects the overall performance of OLEDs. The changes in electrical properties are evident from the difference in current density of OLEDs at the same voltage. The increase in current density for devices annealed at higher temperatures has been observed in other polymer systems^10^,^12^,^25^. Even though the current density of our Super Yellow OLEDs at the same voltage increases with increasing annealing temperature, the efficiency starts to fall after annealing at 50 °C. This is related to the changes observed in thermal and optical properties upon annealing Super Yellow. Efficiency of an OLED is dependent on PL efficiency^3^ and our results show that the PL intensities of Super Yellow films decreased for annealed films. The reduction in PL intensity is directly related to the degradation of polymer with annealing temperature. We see from Fig. 3(a) that the biggest drop in PL intensity is between the non-annealed film and the film annealed at 50 °C. This corresponds to the first weight loss region in TGA of Fig. 3(c), which has the steepest slope. The PL intensity continues to drop for films annealed up to 150 °C. This corresponds to the second region identified in TGA, which has a slower rate of weight loss as compared to the first region. There no noticeable difference in the PL intensity of films annealed at 150 °C and 200 °C. TGA suggests that the polymer degradation is much slower in this temperature range, consistent with the negligible difference in the PL intensities.

The DSC scan revealed a glassy transition for Super Yellow at ~83 °C. Though this T_g_ was determined for the bulk polymer, we expect the T_g_ of thin films in this study to be similar. The films used in this study had a thickness of 90 nm, which is in a film thickness range where T_g_ of polymer films approaches bulk properties^27^,^28^. Once the polymer film is heated beyond its T_g_, the polymer chains have increased mobility and may aggregate. However, from the shapes of the PL spectra of Super Yellow films, we see no signature of any aggregation even when the films are annealed at 200 °C. Normalised PL spectra are shown in Fig. S3 of Supplementary Information. The long side chains of polymer Super Yellow restrict the polymer chains from aggregation. Aggregation of polymer chain is observed in PPV polymers with shorter side chains^12^. The lack of aggregation of the polymer chains in Super Yellow films is also evident from the AFM images of polymer films, which reveal no discernible surface morphological changes with annealing temperature. This property makes Super Yellow OLEDs highly colour stable as is seen in the shape of EL and CIE colour co-ordinates.

In conclusion, we have demonstrated that the annealing temperature for Super Yellow films directly affects the overall performance of OLEDs. OLEDs with annealed Super Yellow films have higher efficiency. However, the maximum current efficiency and EQE dropped from 4.09% to 2.72% and 12.03 cd/A to 8.09 cd/A, respectively, when annealing temperature was increased from 50 °C to 200 °C. The difference in efficiencies observed is related to the thermal degradation and glass transition of the emissive material and not with the morphology of the emissive layer. We believe that the difference in efficiencies will be more pronounced in optically enhanced OLEDs such as microcavity OLEDs, which is a subject of further studies in our group.

## Methods

OLED devices were fabricated on pre-patterned ITO substrates (Kintec). The ITO substrates were cleaned using Alconox and de-ionized water. The substrates were rinsed several times with de-ionised water before ultra-sonicating in acetone and isopropanol consecutively for 10 minutes each. The substrates were dried by blowing with compressed air. PEDOT:PSS (Heraeus) filtered using a 0.45 μm PVDF filter was spin coated on the ITO substrates at 5000 rpm for 30 seconds using a Laurell Technologies spin coater. After removing PEDOT:PSS from the contact pads, the films were annealed at 125 °C for 20 minutes to completely dry the film. The films were then transferred to a glove box system with low moisture and oxygen (O_2_ < 0.1 ppm, H_2_O < 0.1 ppm). Super Yellow solution in anhydrous toluene was prepared a day earlier and kept stirring at room temperature to ensure the polymer is dissolved completely. The PEDOT:PSS films were spin coated with Super Yellow solution using a Speciality Coasting Systems spin coater at 1500 rpm for 30 seconds to obtain a thickness of ~90 nm. Once Super Yellow films were removed from the contact pads, the films were divided into five sets. One set was kept aside while one set each of the remaining four sets were annealed at 50 °C, 100 °C, 150 °C and 200 °C for 20 minutes each. This was followed by vacuum thermal evaporation of 6 nm of Ba (Sigma Aldrich) and 80 nm of Ag (Sigma Aldrich), without breaking vacuum, using a torpedo thermal evaporator at pressures ~10^−6^ mbar. Each OLED pixel had an area of 10 mm^2^. The devices were encapsulated using customized glass caps and UV curable epoxy (NOA 61, Norland Products). Current-Voltage-Luminance (IVL) of the devices were measured using a sourcemeter (B2901A, Keysight Technologies) interfaced with a luminance meter (CS-200, Konica Minolta). The electroluminescence spectra of the devices were recorded using a UV-vis spectrometer (USB4000, Ocean Optics). EQE of the devices were calculated using methods for a Lambertian emitter.

AFM images were taken using an NT-MDT Solver in non-contact mode. Three to five images were acquired on different image areas on different days for each sample, using both small (<5 micron) and large (>5 micron) image areas. Images were processed by using the WSxM software^29^ for background subtraction. WSxM was also used to measure the RMS roughness of the surfaces. Photoluminescence spectra and intensity of Super Yellow films and thickness measurements of all films were done using a Varian fluorescence spectrophotometer (Cary Eclipse) with an excitation wavelength of 400 nm and Bruker Dektak XT profilometer, respectively. Thermal analysis was performed using a Pegasus Q500TGA thermogravimetric analyser under a nitrogen atmosphere at a heating rate of 5 °C/min. Differential scanning calorimeter (DSC) was conducted under nitrogen using a Chimaera Q100 DSC. The sample was heated at 10 °C/min from 25 °C to 300 °C. Gel Permeation Chromatography (GPC) against polystyrene standards was performed in chloroform at 30 °C and a flow rate of 1 mL/min on a Waters GPC assembly equipped with a Waters 1515 isocratic HPLC pump, Waters 2707 autosampler with a 100 mL injection loop, and a Waters 2487 dual wavelength absorbance detector analysed at 254 nm in series with a Waters 2414 refractive index detector at 30 °C. Three consecutive Waters Styragel columns (HR5, HR4, and HR1, all 7.8x´−300 mm, 5 μm particle size), preceded by a Waters Styragel guard column (WAT054405, 4.6x´−30 mm, 20 μm particle size) were used during analysis. A typical concentration of 1 mg polymer dissolved in 1 mL tetrahydrofuran was used to run GPC samples.

## Additional Information

**How to cite this article:** Burns, S. *et al*. Effect of thermal annealing Super Yellow emissive layer on efficiency of OLEDs. *Sci. Rep.* **7**, 40805; doi: 10.1038/srep40805 (2017).

**Publisher's note:** Springer Nature remains neutral with regard to jurisdictional claims in published maps and institutional affiliations.

## Supplementary Material

Supplementary Information
