# Supplementary material for: Effect of thermal annealing Super Yellow emissive layer on efficiency of OLEDs
Source: Sci Rep. 2017 Jan 20;7:40805. doi: 10.1038/srep40805 (PMC5247766; doi:10.1038/srep40805)
Supplement: Supplementary Information [file srep40805-s1.pdf]

Supplementary Information

Effect of thermal annealing Super Yellow emissive layer on efficiency of OLEDs

Samantha Burns[1], Jennifer MacLeod[1,2], Thu Trang Do[1], Prashant Sonar[1], Soniya D. Yambem[1,3,*]

[1]*School of Chemistry Physics and Mechanical Engineering, Queensland University of Technology (QUT), Brisbane QLD 4000, Australia*
[2]*Institute for Future Environments, Queensland University of Technology (QUT), Brisbane QLD 4000, Australia*
[3]*Institute of Health and Biomedical Innovation, Queensland University of Technology (QUT), Kelvin Grove, Queensland 4053, Australia*

Table S1. Different annealing temperatures of polymer Super Yellow thin films in fabrication of organic light emitting devices. OLED: Organic Light Emitting diode, OFET: Organic field effect transistor.

Sl. No.	Temperature (°C)	Time	Light Emitting Device
1.	200	30 mins	OFET[1]
2.	175	30 mins	OFET[2]
3.	140	30 mins	OLED[3]
4.	120	--	OLED[4]
5.	120	--	OLED[5]
6.	100	60 mins	OFET[6]
7.	100	10 mins	OLED[7]
8.	80	30 mins	OLED[8]
9.	80	30 mins	OLED[9]
10.	70	30 mins	OLED[10]
11.	50	15 mins	OLED[11]
12.	--	--	OLED[12]

Table S2. Averages of current efficiency and EQE at different luminance values, averages of maximum current efficiency and EQE and average turn on voltages. The standard deviation is given as error. Highest values are in brackets.

Annealing Temperature	At 100 cd/m^2		At 1000 cd/m^2		At 10000 cd/m2		Maximum		Turn on Voltage
	cd/A	EQE (%)	cd/A	EQE (%)	cd/A	EQE (%)	cd/A	EQE (%)	(V)
Non Annealed	7.96 ± 1.82 (9.32)	2.65 ± 0.61 (3.11)	9.30 ± 1.82 (10.68)	3.11 ± 0.61 (3.57)	8.38 ± 1.23 (9.25)	2.80 ± 0.42 (3.11)	9.40 ± 1.71 (10.73)	3.13 ± 0.57 (3.58)	2.25 ± 0.15
50 °C	9.37 ± 1.03 (10.51)	2.96 ± 0.50 (3.57)	10.14 ± 1.34 (11.90)	3.22 ± 0.58 (4.04)	8.92 ± 1.06 (10.18)	2.87 ± 0.44 (3.47)	10.37 ± 1.30 (12.03)	3.52 ± 0.44 (4.09)	2.16 ± 0.05
100 °C	4.38 ± 1.82 (6.96)	1.49 ± 0.62 (2.38)	7.05 ± 1.89 (9.87)	2.40 ± 0.66 (3.39)	8.49 ± 1.58 (10.27)	2.89 ± 0.55 (3.49)	8.66 ± 1.76 (10.69)	2.97 ± 0.60 (3.67)	2.28 ± 2.17

**150 °C**	6.73 ± 0.83 (7.71)	2.27 ± 0.29 (2.62)	8.73 ± 1.30 (10.21)	2.94 ± 0.43 (3.44)	8.17 ± 1.25 (9.63)	2.76 ± 0.43 (3.27)	9.04 ± 1.47 (10.37)	2.96 ± 0.45 (3.48)	2.16 ± 0.08
**200 °C**	4.02 ± 0.32 (4.34)	1.34 ± 0.11 (1.43)	6.10 ± 0.71 (6.76)	2.04 ± 0.24 (2.27)	6.71 ± 1.13 (7.80)	2.24 ± 0.37 (2.60)	7.31 ± 0.76 (8.07)	2.31 ± 0.38 (2.72)	2.10 ± 0.05

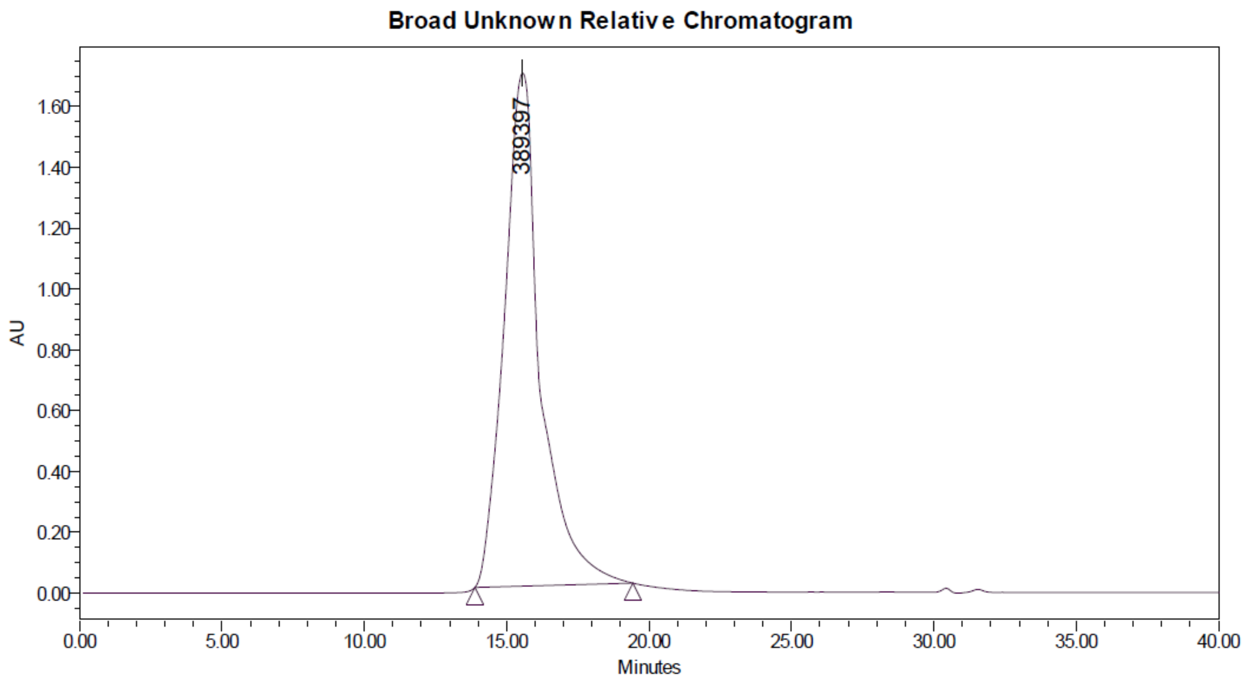

Broad Unknown Relative Peak Table

	Distribution Name	Mn (Daltons)	Mw (Daltons)	MP (Daltons)	Mz+1 (Daltons)	Polydispersity	Mz/Mw	Mz+1/Mw
1		184300	254594	389397	322830	1.381408	1.168589	1.268018

Figure S1. Gel permeation chromatogram of the super yellow polymer in tetrahydrofuran at 30 °C. M_R are relative to polystyrene standards.

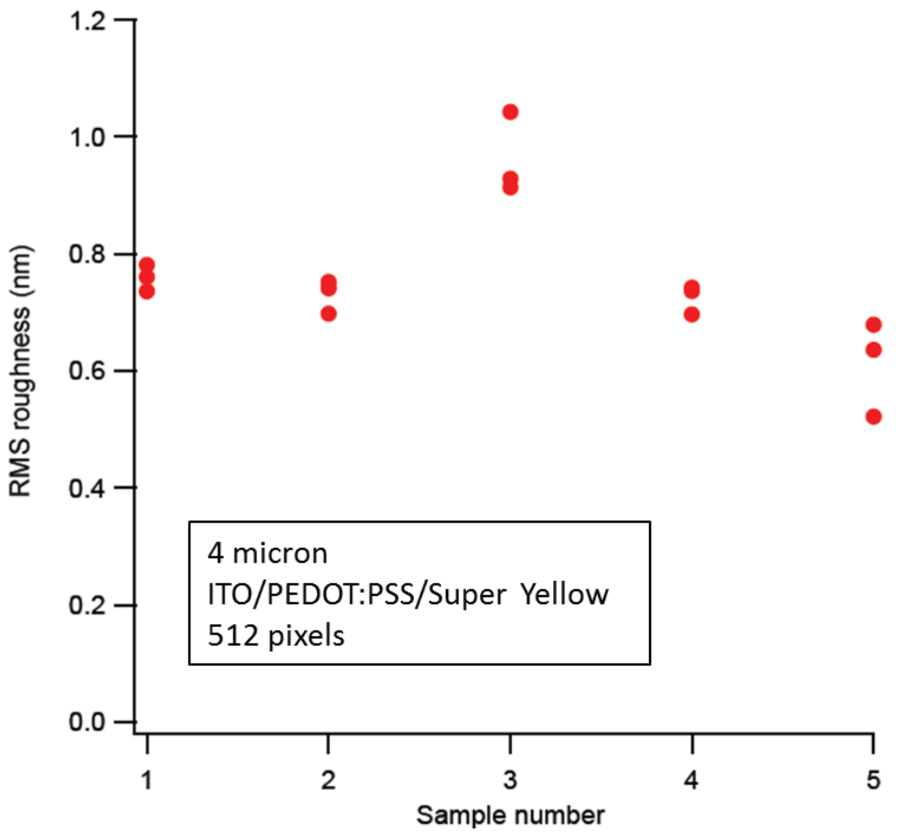

Figure S2. RMS roughness of ITO/PEDOT:PSS/Super Yellow films on glass annealed at different temperatures. Sample numbers 1, 2, 3, 4 and 5 correspond to non-annealed, annealed at 50 ˚C, 100 ˚C, 150 ˚C and 200 ˚C, respectively.

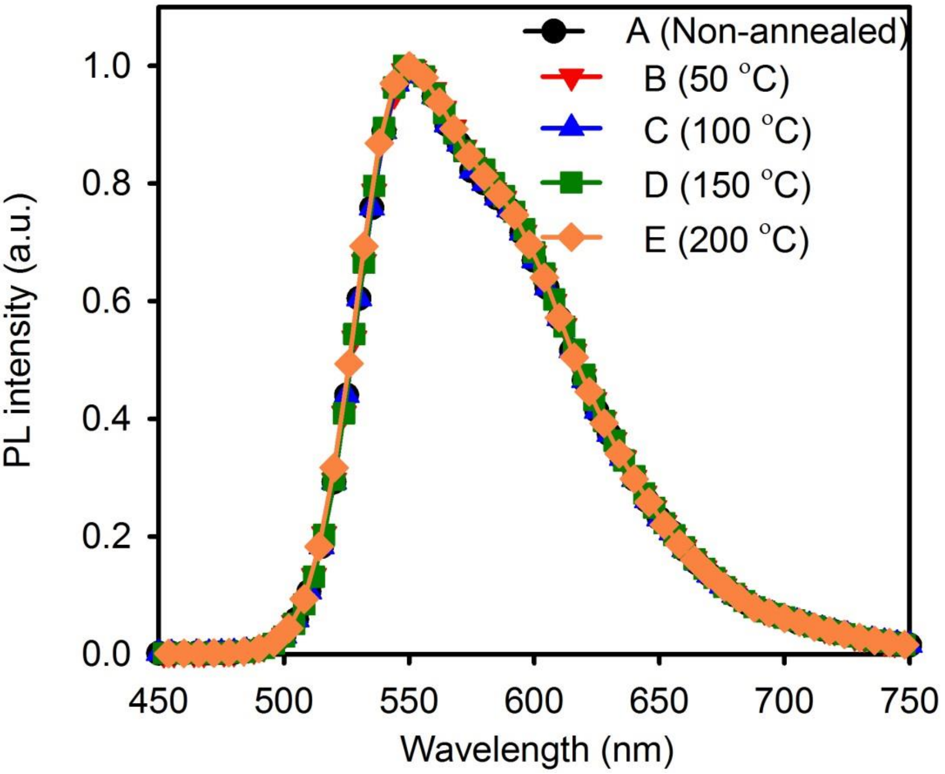

Figure S3. Normalized PL spectra for non-annealed and annealed Super Yellow thin films.

References:

1 Namdas, E. B., Ledochowitsch, P., Yuen, J. D., Moses, D. & Heeger, A. J. High performance light emitting transistors. *Applied Physics Letters* **92,** 183304 (2008).

2 Namdas, E. B. *et al.* Gate-Controlled Light Emitting Diodes. *Advanced Materials* **20,** 1321-1324 (2008).

3 Cha, S. J. *et al.* Thermally Cross-Linkable Hole Transport Polymers for Solution-Based Organic Light-Emitting Diodes. *Macromolecular Rapid Communications* **35,** 807-812 (2014).

4 Tseng, S.-R., Meng, H.-F., Lee, K.-C. & Horng, S.-F. Multilayer polymer light-emitting diodes by blade coating method. *Applied Physics Letters* **93,** 153308 (2008).

5 Tseng, S. R. *et al.* Electron transport and electroluminescent efficiency of conjugated polymers. *Synthetic Metals* **159,** 137-141 (2009).

6 Kane-Maguire, L. A. P., Officer, D. L., Swensen, J., Moses, D. & Heeger, A. J. Proceedings of the International Conference on Science and Technology of Synthetic MetalsLight emission in the channel region of a polymer thin-film transistor fabricated with gold and aluminum for the source and drain electrodes. *Synthetic Metals* **153,** 53-56 (2005).

7 Calvin Yi Bin, N. *et al.* High efficiency solution processed fluorescent yellow organic light-emitting diode through fluorinated alcohol treatment at the emissive layer/cathode interface. *Journal of Physics D: Applied Physics* **47,** 015106 (2014).

8 Lee, B. R. *et al.* Highly Efficient Polymer Light-Emitting Diodes Using Graphene Oxide as a Hole Transport Layer. *ACS Nano* **6,** 2984-2991 (2012).

9 Ko, S.-J. *et al.* Highly efficient plasmonic organic optoelectronic devices based on a conducting polymer electrode incorporated with silver nanoparticles. *Energy & Environmental Science* **6,** 1949-1955 (2013).

10 Hassan, M. U. *et al.* Highly efficient PLEDs based on poly(9,9-dioctylfluorene) and Super Yellow blend with Cs2CO3 modified cathode. *Applied Materials Today* **1,** 45-51 (2015).

11 Gilissen, K. *et al.* Ultrasonic spray coating as deposition technique for the light-emitting layer in polymer LEDs. *Organic Electronics* **20,** 31-35 (2015).
12 Pradana, A. & Gerken, M. Photonic crystal slabs in flexible organic light-emitting diodes. *Photon. Res.* **3,** 32-37 (2015).
